# Granulomatosis with Polyangiitis with Bilateral Facial Palsy and Severe Mixed Hearing Loss

**DOI:** 10.1155/2016/5206170

**Published:** 2016-07-14

**Authors:** Agnieszka Wawrzecka, Anna Szymańska, Radosław Jeleniewicz, Marcin Szymański

**Affiliations:** ^1^Department of Otolaryngology Head and Neck Surgery, Medical University of Lublin, Jaczewskiego 8, 20-954 Lublin, Poland; ^2^Department of Neuroradiology and Interventional Radiology, Medical University of Lublin, Jaczewskiego 8, 20-954 Lublin, Poland; ^3^Department of Rheumatology, Medical University of Lublin, Jaczewskiego 8, 20-954 Lublin, Poland

## Abstract

Granulomatosis with polyangiitis is autoimmune and rare disease. It affects many organs, but the most often affected organs are the nose, lungs, and kidneys. It is part of vasculitis and causes an autoimmune attack by an abnormal type of circulating antibody termed ANCAs against small blood vessels. Disease concerns both men and women with a peak age of presentation in the sixth and seven decades. Typically upper and lower respiratory tract and kidneys are involved. Otitis externa, otitis media, or mastoiditis rarely occurs in granulomatosis with polyangiitis. Deafness is the most dangerous aural complication. Histological examination of biopsy is often not specific. A case of GPA with bilateral otitis media, bilateral deafness, and bilateral facial palsy with fatal course is presented.

## 1. Introduction

Granulomatosis with polyangiitis (GPA) is a rare, autoimmune, multisystemic disease first described by Friedrich Wegener in 1936 [1, 2] and has been named Wegener's granulomatosis for many years. In 2012, the name Wegener's granulomatosis (WG) was adopted by the 2012 International Chapel Hill Consensus Conference and it is now named granulomatosis with polyangiitis. GPA is necrotizing granulomatous inflammation involving the upper and lower respiratory tract and necrotizing vasculitis affecting predominantly small-to-medium vessels. Necrotizing glomerulonephritis is common. GPA belongs to AAV (ANCA-associated vasculitis) [3].

GPA is a relatively rare condition, with a peak age of presentation in the sixth and seven decades of life. However, it can appear at any age, with no gender predilection [4]. The prevalence of the disease ranges between 12 cases per million inhabitants per year in Norway and 3 cases per million inhabitants in Spain [5, 6]. Typically upper and lower respiratory tract and kidneys are involved. The diagnostic criteria established by the American College of Rheumatology in 1990 include the following: hematuria, abnormal chest radiograph, ulceration in the mouth and/or nose, and positive histopathological evaluation [7]. To confirm the diagnosis of GPA, two of these have to be stated. Approximately one-third of patients may present with a limited, locoregional form of the disease, without renal involvement [8]. Otologic symptoms may be present in the course of the disease but rarely are the first to appear [9–11]. Facial paralysis and deafness as primary manifestation of GPA are uncommon. We present a rare case of progressive GPA with unusual presentation and fatal course.

## 2. Case Report

A 56-year-old female presented with bilateral otalgia and hypoacusis gradually progressing for the past two weeks and left sided facial palsy significantly increasing within two days. She has been previously twice unsuccessfully treated with antibiotics in another hospital due to chronic otitis media. She also had a history of psoriasis and hyperthyreosis. AAV may occur with antithyroid drug therapy. However, the patient was treated only one month before the blood test; thus the possibility of drug-induced AAV is low.

Otoscopic examination revealed bilaterally thickened and reddish eardrums. There was a subtotal perforation of the right tympanic membrane and an anterior perforation of the left tympanic membrane with effusion. Left facial nerve palsy was categorized as grade IV according to House-Brackmann. Nose examination was normal. The pure tone audiometry showed severe bilateral mixed hearing loss on the level of 80–100 dB with air bone. On the right the threshold is on the level of 95–100 dB with air bone gap of 50 dB. On the left the threshold gap is 70 dB. Chest X-ray revealed signs of bronchitis. Urine analysis was normal.

**Figure 1:**
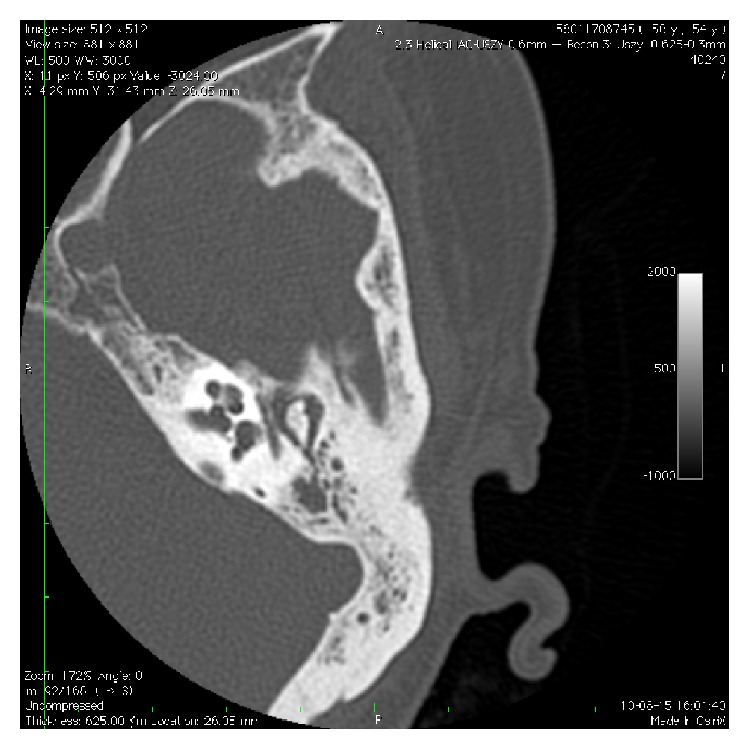
CT scan shows sclerotic left mastoid process and oppacified tympanic cavity.

High resolution computed tomography scans of the temporal bones showed bilateral sclerosing mastoiditis and opacification of the right tympanic cavity with an air-fluid level. No signs of bony destruction within the ossicular chain, the internal ear, or facial canal were present (Figure 1). Magnetic resonance imaging (MRI) with the use of T2-weighted, T1-weighted, and contrast enhanced images was performed and showed normal appearance of cerebral structures and mastoid cells filled with fluid on both sides. Hypertrophic pachymeningitis sometimes shows in the GPA patients wit hear involvement. In this case there were no signs of pachymeningitis on MRI examination. The diagnosis of acute otitis media with peripheral facial nerve paralysis was made and intravenous antibiotics treatment was started. As there was no response to drug therapy, the patient was referred to myringoplasty and antromastoidectomy with facial nerve decompression. During surgery, granulation in the mastoid cavity was found. There was no granulation on the facial nerve canal. The facial nerve was pale and swollen during decompression. After surgery otalgia and hearing on the left side improved slightly, but facial paralysis did not show any improvement. The patient was discharged and sent home with prescribed antibiotic treatment.

After two months, the patient's condition deteriorated significantly as she presented with bilateral facial paresis (House-Brackman grades IV-V), horizontal nystagmus directed to the right ear, severe hearing loss (70–80 dB), otalgia, and otorrhea. Blood tests showed WBC of 12,93 thousand/*μ*L (normal range: 4–10) and CRP of 147,76 mg/L (normal range: 5,0). Laboratory tests showed hyperthyreosis with positive results for antibody to thyroglobulin: A-TG of 180 U/L (normal range: 0,0–60). Renal function tests and abdominal ultrasound were normal. Intravenous antibiotic treatment was administered.

**Figure 2:**
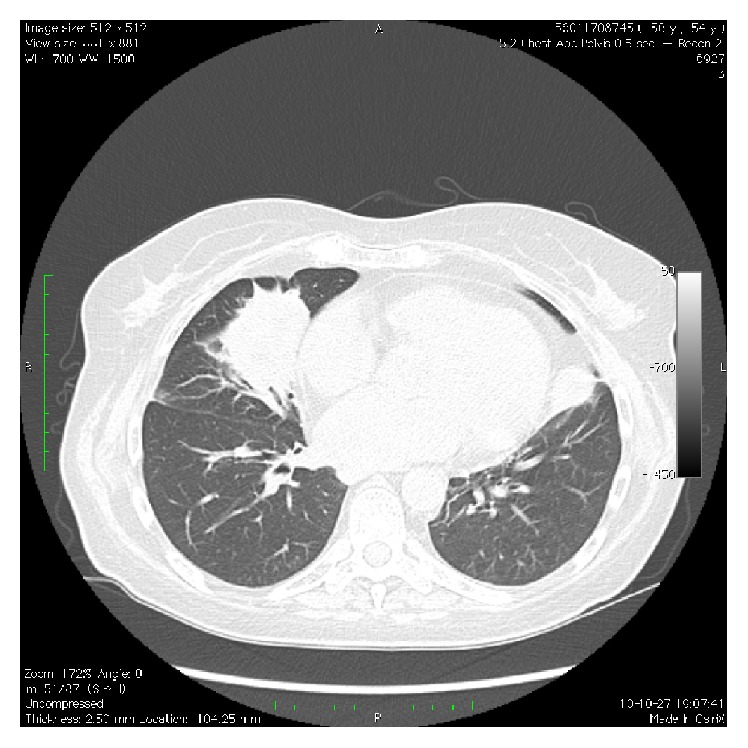
CT of the chest shows two metastatic focuses in both lungs and mediastinal nodules.

Chest X-ray showed bilateral hilar mass lesions. Chest CT scans revealed mediastinal nodules and parenchymal consolidations in both lungs (Figure 2). Head MRI showed no pathological changes except for complete opacification of mastoid cells and middle ears bilaterally. Ophthalmic evaluation revealed bilateral scleritis.

Due to the suspicion of lungs metastatic disease, the patient was referred to thoracotomy. The histopathology tests of pulmonary lesion suggested tuberculosis, which resulted in introduction of tuberculostatic treatment. With no improvement after tuberculostatic treatment and suspicion of GPA, serological ELISA tests were performed and c-ANCA tests were positive. Subsequently the patient was transferred to Rheumatology Department, where the diagnosis of GPA was confirmed. Control laboratory tests showed WBC of 14,9 thousand/*μ*L (normal range: 4–10), markedly increased value of c-ANKA > 150 U/L (>8,0 U/mL positive result), and abnormal urine analysis with the presence of protein and red blood cells. Follow-up head CT showed chronic inflammatory changes with polyps in paranasal sinuses.

The patient was commenced on systemic steroid therapy in the form of Solu-Medrol 2,0 g per day and cyclophosphamide (Endoxan) 600 mg per day with the protection of Uromitexan. The total dose of Endoxan was 5,4 g. In the course of treatment, the patient's condition improved concerning hearing and nystagmus. Facial nerve palsy regressed from 5th to 2nd grade bilaterally in House-Brackman scale.

Unfortunately, the patient's renal function deteriorated gradually and she died 2 years after the initial otological symptoms due to progressive renal disease.

## 3. Discussion

The clinical manifestation of GPA is heterogeneous. The typical triad consists of the upper respiratory tract, lungs, and kidneys involvement. Head and neck involvement in the initial phase of GPA is not uncommon, accounting for 70–95% of cases [12, 13]. It was observed in 72% of 411 patients evaluated by McCaffrey et al. [10]. Morales-Angulo et al. reported ENT manifestations in the course of the disease in 88% of patients, and in 28% of cases it was the first symptom of the disease. Several head and neck regions may be affected by the disease, with nose and paranasal sinuses being the most frequently involved sites (60–90% of cases) [13, 14]. The GPA lesions may also be located in the larynx, oral cavity, orbit, and parotid gland [13].

The prevalence of ear disorders varies from 19% to 70% of cases [10, 13]. Rarely, otological symptoms may be the presenting signs of GPA. Bakthavachalam et al. [15] reported that in 14% of patients with GPA hearing loss preceded the correct diagnosis. The etiology of sensorineural hearing loss (SNH) in patients with GPA is unclear. It occurs in approximately 43% of patients and is thought to be associated with vasculitis of cochlea vessels, with compression of the cochlea nerve by the granulomatous tissue, or with the deposits of immune complexes in the labyrinth inducing labyrinthitis [7]. Vertigo is rare and may develop as a result of similar mechanisms as SNH.

Most common otologic disorders in GPA patients are middle ear lesions (40–70% of cases). Unilateral serous otitis media is the most frequent manifestation, present in 90% of cases, and bilateral otitis media occurs in 33% of cases [16]. In 90% of cases unilateral or bilateral middle ear disease develops secondary to the formation of granulation tissue in the nasopharynx and the area of the Eustachian tube, which results in secretory changes [17]. Chronic otitis media is associated with the presence of granulation tissue in the middle ear and the mastoid, which occurs in approximately 24% of cases [16, 17]. Destructive granulomatous masses may cause erosion of the ossicles or spread through the mastoid towards the petrous apex [18]. This process may be accompanied by effusion, mastoiditis, and/or facial palsy [14]. Osteomastoiditis accompanied by peripheral facial nerve palsy may occur in 8%–10% of cases [14, 16].

However, facial palsy as the first manifestation of GPA, like in our patient, is very rare and only few cases have been reported in the literature [16]. Moreover, the combination of bilateral advanced sensorineural hearing loss and unilateral facial palsy as presenting signs of the disease is very uncommon [16]. Occasionally, the progressive course of the disease may lead to bilateral facial palsy, which was observed in our patient. Bilateral facial palsy is an extremely rare finding [19]. In the review of 2856 patients with facial paralysis Lee et al. reported only 2% of cases with bilateral involvement [20]. In the course of GPA with bilateral ears disorders, bilateral facial palsy develops extremely rare with only few cases reported in the literature [20].

The diagnosis of GPA is performed on the basis of the characteristic clinical presentation, that is, head and neck symptoms and signs typically accompanied by pulmonary and/or renal disease. Suggestive histopathological study showing the presence of granulomatous inflammation with necrosis, multinucleated cells, and vasculitis confirms the diagnosis. However, histopathological identification of GPA may be difficult, especially in the locoregional form of the disease. Biopsy sample of the granulation tissue from the middle ear is often of limited amount and may show nonspecific granulomatous disease. In our patient, histopathological examination of the pulmonary lesion was suggestive for tuberculosis, which precluded a correct diagnosis. Morales-Angulo et al. [12] reported that in 50% of patients who underwent head and neck biopsy of suspicious lesions (mainly nasal) the result confirmed the GPA by showing typical histological criteria. Other authors confirm that often multiple biopsies are required, because the results are inconclusive in over 50% of cases [14]. Bradley [21] suggested that to make a diagnosis of GPA complete histopathological picture is not always essential. With suggestive clinical manifestation of the disease, lack of typical histopathological picture is acceptable.

Introduction of tests determining serum levels of specific markers such as antineutrophil cytoplasmic antibodies (ANCA) and PR3 revolutionized the diagnosis of GPA, since they allow early diagnosis [16, 18]. The specificity of positive c-ANCA testing in GPA is greater than 95% [20]. The levels of c-ANCA tests correlate well with the disease activity, falling in patients during proper treatment and rising if a relapse after treatment occurs [18, 20]. The sensitivity of c-ANCA tests in the generalized form of the disease is 93%–97%, whereas in the regional form it is approximately 60% [16, 18]. Positive ANCA tests may help in setting the correct diagnosis in histologically dubious cases, which was the case in our patient.

The standard treatment for patients with GPA is a combination of immunosuppressive drugs and steroids [16]. It is recommended to use such therapy also in patients with head and neck manifestations of GPA.

Prognosis of neuropathies in the course of GPA depends on the early correct diagnosis and proper timely treatment. Many authors recommend conservative treatment, as it usually results in nerve function recovery, whereas surgery may increase the risk of additional nerve injury [14, 19]. However, unusual manifestation at the onset of the disease may delay the correct diagnosis and the suspicion of GPA only appears in the case of failure of conventional therapy or when additional symptoms from other organs occur. It is suggested that prolonged evolution of over 20 days to observe regression of ear inflammation raises a suspicion of a specific etiology responsible for disease activity [14]. Our patient underwent surgery at the initial phase of the disease. Fortunately, surgical intervention in our patient did not cause further deterioration of facial nerve function.

The course of the GPA may be variable. If untreated, the disease may be lethal. In such case, 80–82% of patients die within 1 year after diagnosis [19]. Approximately 25% of patients present a fulminant course of the disease with multiple organs involvement and subsequent failure [2]. Progressive renal disease is the most common cause of death. Our patient evolved this way, with the disease running rapidly fatal course leading to death within 2 years from the initial otological symptoms. Preuss et al. [9] presented similar case of patient with GP-induced bilateral facial palsy together with renal and pulmonary involvement, which despite intensive treatment had a fatal course due to multiorgan failure [19]. The average survival of patients with GPA is 5 months [19]. However, prompt initiation of suitable treatment may result in long-term remissions in up to 90% of patients, especially before renal involvement [14].

## 4. Conclusions 

In patients with GPA, facial nerve palsy may develop not only in a classical form of a disease subsequent to middle ear involvement but also occasionally in a locoregional form as primary otological manifestation accompanied by sensorineural hearing loss. In such cases, otolaryngologists have a crucial role in recognizing the early symptoms of the disease and starting the appropriate therapy. Otitis media failing in responding to conventional treatment, facial nerve palsy accompanying the middle ear disease, or symptoms from other organs suggesting the multisystemic involvement should always raise a suspicion of GPA. Positive ANCA tests contribute to correct diagnosis in dubious cases. Early diagnosis may prevent the unnecessary and potentially hazardous surgical treatment and reduce the mortality and morbidity associated with the disease.

## References

[1] Falk R. J., Gross W. L., Guillevin L. (2011). Granulomatosis with polyangiitis (Wegener's): an alternative name for Wegener's granulomatosis. *Arthritis and Rheumatism*.

[2] Wierzbicka M., Puszczewicz M., Bartochowska A., Szyfter W. (2012). The otologic manifestation of Wegener's granulomatosis—review of contemporary achievements in diagnostics and treatment. *Otolaryngologia Polska*.

[3] Jennette J. C., Falk R. J., Bacon P. A. (2013). 2012 Revised International Chapel Hill consensus conference nomenclature of vasculitides. *Arthritis & Rheumatism*.

[4] González-Gay M. A., García-Porrúa C. (2001). Epidemiology of the vasculitides. *Rheumatic Disease Clinics of North America*.

[5] Gonzalez-Gay M. A., Garcia-Porrua C., Guerrero J., Rodriguez-Ledo P., Llorca J. (2003). The epidemiology of the primary systemic vasculitides in northwest Spain: implications of the Chapel Hill Consensus Conference definitions. *Arthritis Care and Research*.

[6] Koldingsnes W., Nossent H. (2000). Epidemiology of Wegener's granulomatosis in northern Norway. *Arthritis and Rheumatism*.

[7] Takagi D., Nakamaru Y., Maguchi S., Furuta Y., Fukuda S. (2002). Otologic manifestations of Wegener's granulomatosis. *Laryngoscope*.

[8] Cassan S. M., Coles D. T., Harrison E. G. (1970). The concept of limited forms of Wegener's granulomatosis. *The American Journal of Medicine*.

[9] Preuss S. F., Stenner M., Beutner D., Laudes M., Klussmann J. P. (2008). Fatal course of Wegener's granulomatosis with bilateral otomastoiditis and bilateral facial nerve palsy. *Otolaryngology—Head and Neck Surgery*.

[10] McCaffrey T. V., McDonald T. J., Facer G. W., DeRemee R. A. (1980). Otologic manifestations of Wegener's granulomatosis. *Otolaryngology-Head and Neck Surgery*.

[11] Kornblut A. D., Wolff S. M., Fauci A. S. (1982). Ear disease in patients with wegener’s granulomatosis. *Laryngoscope*.

[12] Morales-Angulo C., García-Zornoza R., Obeso-Agüera S., Calvo-Alén J., González-Gay M. A. (2012). Ear, nose and throat manifestations of Wegener's granulomatosis (granulomatosis with polyangiitis). *Acta Otorrinolaringológica Española*.

[13] Gottschlich S., Ambrosch P., Kramkowski D. (2006). Head and neck manifestations of Wegener's granulomatosis. *Rhinology*.

[14] Maranhão A. S. D. A., Chen V. G., Rossini B. A. A., Testa J. R. G., Penido N. D. O. (2012). Mastoiditis and facial paralysis as initial manifestations of Wegener's Granulomatosis. *Brazilian Journal of Otorhinolaryngology*.

[15] Bakthavachalam S., Driver M. S., Cox C., Spiegel J. H., Grundfast K. M., Merkel P. A. (2004). Hearing loss in Wegener's granulomatosis. *Otology and Neurotology*.

[16] Verma N., Gupta A. (2012). Wegener's granulomatosis: an unusual presentation case report and review of the literature. *The Internet Journal of Otorhinolaryngology*.

[17] Ferri E., Armato E., Capuzzo P., Cavaleri S., Ianniello F. (2007). Early diagnosis of Wegener's granulomatosis presenting with bilateral facial paralysis and bilateral serous otitis media. *Auris Nasus Larynx*.

[18] Sharma A., Deshmukh S., Dabholkar J. (2013). ENT manifestations of Wegeners granulomatosis. *Otolaryngologia Polska*.

[19] Roszkowska A., Morawska-Kochman M., Temporale H., Sikorska-Żuk M., Kręcicki T. (2013). Bilateral facial palsy in rapidly progressive course of wegener’s granulomatosis: a case report. *Case Reports in Otolaryngology*.

[20] Lee J. H., Kim K. W., Myong N. H., Jung J. Y. (2013). Wegener's granulomatosis presenting as bilateral otalgia with facial palsy: a case report. *Korean Journal of Audiology*.

[21] Bradley P. J. (1983). Wegener's granulomatosis of the ear. *The Journal of Laryngology & Otology*.

